# Beneficial effects of miR-132/212 deficiency in the zQ175 mouse model of Huntington’s disease

**DOI:** 10.3389/fnins.2024.1421680

**Published:** 2024-08-07

**Authors:** Behnaz Nateghi, Remi Keraudren, Gabriel Boulay, Marc Bazin, Claudia Goupil, Geoffrey Canet, Andréanne Loiselle, Isabelle St-Amour, Emmanuel Planel, Denis Soulet, Sébastien S. Hébert

**Affiliations:** ^1^Axe Neurosciences, Centre de Recherche du CHU de Québec-Université Laval, CHUL, Québec, QC, Canada; ^2^Département de Psychiatrie et de Neurosciences, Faculté de Médecine, Université Laval, Québec, QC, Canada; ^3^CERVO Brain Research Centre, Centre Intégré Universitaire de Santé et des Services Sociaux de la Capitale-Nationale, Québec, QC, Canada; ^4^Faculté de Pharmacie, Université Laval, Québec, QC, Canada

**Keywords:** Huntington’s disease, miR-132/212 cluster, knockout mice, RNA-seq, neurodegeneration

## Abstract

Huntington’s disease (HD) is a rare genetic neurodegenerative disorder caused by an expansion of CAG repeats in the Huntingtin (HTT) gene. One hypothesis suggests that the mutant HTT gene contributes to HD neuropathology through transcriptional dysregulation involving microRNAs (miRNAs). In particular, the miR-132/212 cluster is strongly diminished in the HD brain. This study explores the effects of miR-132/212 deficiency specifically in adult HD zQ175 mice. The absence of miR-132/212 did not impact body weight, body temperature, or survival rates. Surprisingly, miR-132/212 loss seemed to alleviate, in part, the effects on endogenous Htt expression, HTT inclusions, and neuronal integrity in HD zQ175 mice. Additionally, miR-132/212 depletion led to age-dependent improvements in certain motor functions. Transcriptomic analysis revealed alterations in HD-related networks in WT- and HD zQ175-miR-132/212-deficient mice, including significant overlap in BDNF and Creb1 signaling pathways. Interestingly, however, a higher number of miR-132/212 gene targets was observed in HD zQ175 mice lacking the miR-132/212 cluster, especially in the striatum. These findings suggest a nuanced interplay between miR-132/212 expression and HD pathogenesis, providing potential insights into therapeutic interventions. Further investigation is needed to fully understand the underlying mechanisms and therapeutic potential of modulating miR-132/212 expression during HD progression.

## 1 Introduction

Huntington’s disease (HD) is a dominantly inherited neurodegenerative disorder caused by a CAG repeat expansion in the first exon of the Huntingtin gene (HTT) (Ross and Shoulson, 2009; Ross and Tabrizi, 2011). The mutant HTT protein is expressed ubiquitously and causes progressive neuronal dysfunction and degeneration that primarily affects the striatum and spreads to other brain regions as the disease progresses. HD is characterized by involuntary choreatic movements with cognitive and behavioral disturbances. While several motor and psychiatric features of HD can be symptomatically treated, a disease-modifying treatment to slow or stop the disease progression is still unavailable. Furthermore, the underlying molecular mechanisms of HD pathology and neurodegeneration remain unresolved.

It has been suggested that mutant HTT promotes neuronal dysfunction through transcriptional dysregulation, one of the earliest and central pathogenic mechanisms observed in HD brain (Ross and Tabrizi, 2011). Among key changes in gene expression pathways in HD include neurotrophins such as BNDF (Zuccato et al., 2001; Canals et al., 2004; Strand et al., 2007). BDNF levels are reduced in HD, whereas introducing BDNF can restore neuronal dysfunction in disease models. Similar findings were obtained by stimulating downstream mediators of BDNF, such as Creb1, Mecp2, and Mapk/ERK (Bodai and Marsh, 2012; McFarland et al., 2014; Abd-Elrahman and Ferguson, 2019). If and how these cascades interconnect *in vivo* in HD still needs to be better understood.

Accumulating evidence suggests that microRNAs (miRNAs) participate in the transcriptional landscape of HD. These short (∼22 nts) non-coding RNAs bind to the 3’ untranslated region (3’UTR) of mRNA targets promoting translation inhibition or degradation. MiRNAs can regulate one or multiple genes within a biological network. Interestingly, the complete abrogation of miRNAs in neurons leads to alterations in transcription, reduced brain size, behavioral defects, and decreased lifespan in mice, as seen in HD (Cuellar et al., 2008; Davis et al., 2008; Dorval et al., 2012). In recent years, several individual miRNAs have been linked to HD pathogenesis (Dong and Cong, 2021; Martinez and Peplow, 2021; Tung et al., 2021). However, no current evidence from *in vivo* knockout studies supports the contribution of specific miRNAs in HD.

We and others have shown that the miR-132/212 cluster is among the most robust miRNA (family) downregulated in HD brain and cell models (Johnson et al., 2008; Lee et al., 2011; Soldati et al., 2013; Fukuoka et al., 2018; Dubois et al., 2021; Petry et al., 2022). Both miR-132 and miR-212 are expressed from the same transcript on chromosome 17 in humans (11 in mice). They have the same seed region important for mRNA binding and share most (if not all) biological targets (Remenyi et al., 2010; Wanet et al., 2012) (targetscan.org). Interestingly, miR-132/212 is functionally connected to numerous BNDF mediators, including Creb1 and Mapk/ERK (Impey et al., 2010; Remenyi et al., 2010; Numakawa et al., 2011; Yi et al., 2014; Keifer et al., 2015; Qian et al., 2017). For instance, miR-132/212 expression is stimulated by BNDF, Creb1, and Mapk/ERK, while the same genes are misregulated in miR-132/212 full-knockout mice (Hernandez-Rapp et al., 2015, 2016; Kouhnavardi et al., 2023). Interestingly, miR-132 overexpression rescues motor deficits in HD mice, possibly involving Mecp2 and yet unidentified factors (Fukuoka et al., 2018).

Despite these findings, the impact of miR-132/212 deficiency on HD neuropathology *in vivo* remains unknown. For this reason, we examined the physiological, biochemical, and transcriptional effects of miR-132/212 knockout in adult heterozygote zQ175 knock-in mice, a popular HD mouse model that develops pathological and clinical-like symptoms during adulthood (Menalled et al., 2012; Carty et al., 2015; Jansen et al., 2017; Smith et al., 2023). To our surprise, we show that removing the miR-132/212 cluster in HD mice seems to restore, at least in part, disease-related phenotypes, highlighting a complex relationship between miR-132/212 regulation and function in HD.

## 2 Materials and methods

### 2.1 Human brain samples

Dissected frozen human putamen, cortical (BA39 and B4 regions), and cerebellum tissues were obtained from the Harvard Brain Tissues Resource Center via the NIH NeuroBioBank (Supplementary Table 1). This study included matching tissues from 10 controls and 10 HD individuals. Frozen post-mortem tissues were prepared as described previously and used for protein and RNA analysis (St-Amour et al., 2018; Petry et al., 2022, 2023).

### 2.2 Animals

The miR-132/212 conditional knockout (cKO) mice (miR-132/212^flox/flox^, a kind gift from Dr. R. H. Goodman, Vollum Institute, USA) (Hernandez-Rapp et al., 2016) were bred with R26-CreERT2 mice (JAX Strain #008463). Cre-positive miR-132/212 cKO mice were then crossed with heterozygote zQ175 (zQ175 −/+) HD knock-in mice containing the human HTT exon 1 sequence with a ∼190 CAG repeat tract (JAX, Strain #027410). To induce widespread miR-132/212 deletion in adults, 2-month-old mice were injected i.p., with 200 μg of tamoxifen for five consecutive days. We generated four experimental subgroups of mice with respective littermate controls, all of which were positive for Cre expression and injected with tamoxifen, namely WT (miR-132/212 +/+, zQ175 −/−), KO (miR-132/212^flox/flox^, zQ175 −/−), HD (miR-132/212 +/+, zQ175 −/+), and HD/KO (miR-132/212^flox/flox^, zQ175 −/+). The mice were housed under a 12:12 h light/dark cycle with food and water *ad libitum*. The injected mice were sacrificed at 18 months of age by decapitation, and the tissues of interest were removed, dissected on ice and frozen on dry ice, as previously described (Hernandez-Rapp et al., 2016). Tissues were stored at −80°C until use. Detailed breeding and genotyping information are available upon request.

### 2.3 Protein and RNA analyses

#### 2.3.1 Protein and RNA extraction

For protein extraction, frozen tissues were mechanically homogenized in five volumes of lysis buffer [150 nM NaCl, 50 mM Tris, 0.5% deoxycholate, 1% Triton X-100, 0.5% sodium dodecyl sulfate (SDS), complete protease inhibitor cocktail, 1 mM of sodium fluoride, 1 mM of activated orthovanadate], then sonicated three times and spun at 20,000 g for 20 min at 4°C. The supernatant (soluble proteins) was kept at −80°C until processed. Total RNA was extracted from frozen tissues using TRIzol reagent (Life Technologies) according to the manufacturer’s instructions. The pellet was dissolved in RNase-free water and quantified by spectrophotometry (Tecan Infinite F200). Total RNA was kept at −80°C until use.

#### 2.3.2 Western blotting

Proteins were quantified with Pierce™ BCA Protein Assay Kit (ThermoFisher Scientific). Five to 20 micrograms of soluble proteins in Laemmli solution containing beta-mercaptoethanol were separated using 10% Tris-Glycine extended (TGX) Stain-Free™ polyacrylamide gels (Bio-Rad, Hercules, CA, USA) or 3% SDS–polyacrylamide gels (SDS-Page) gels for HTT protein. The Stain-Free™ gels were activated by UV transillumination for 5 min using the Fusion FX5 imaging system (Vilbert Lourmat, France). Proteins were transferred onto a 0.45 μm nitrocellulose membrane (Bio-Rad, catalog n° 1620115) for 1 h at 100 V, and total proteins were visualized under UV using the Fusion FX5 imaging system. For the 3% SDS-Page gels, proteins were transferred onto a 0.45 μm methanol-activated PVDF membrane (Immobilon, Millipore) overnight at 4°C at 25 V and 45 min at 4°C at 75 V the next day. The membrane was blocked with 5% non-fat milk and incubated overnight at 4°C with the appropriate primary antibodies (Supplementary Table 2). On the second day, membranes were incubated with appropriate secondary anti-IgG-HRP antibodies (Jackson ImmunoResearch: anti-mouse, catalog no 115-035-146 or anti-rabbit, catalog no 111-035-144) 1:5000 in 5% milk at RT for 1 h. The immune-reactive bands were acquired using Immobilon Western Chemiluminescent HRP Substrate (Millipore) and visualized with the ImageQuantTM LAS 4000 (GE Healthcare Bio-Sciences AB) imaging system. Normalization was performed on the total amount of protein per lane based on Ponceau or Stain-Free staining. Band intensities were quantified using the ImageJ software.

#### 2.3.3 microRNA real time quantitative RT-PCR

The reverse transcription was performed with 10 ng of total RNA using the TaqMan MicroRNA Reverse transcription kit (ThermoFisher) according to the manufacturer’s instructions (Program: 16°C for 30 min, 42°C for 30 min and 85°C for 5 min). The qRT-PCR was performed with TaqMan Fast Advanced Master mix (ThermoFisher Scientific) according to the manufacturer’s instructions. miRNA assay primers were purchased from ThermoFisher Scientific (hsa-miR132, 000457; hsa-miR212, 000515). Normalization was performed on the geographic mean of RNU48 (hsa-RNU48, 001006) and Let-7f (hsa-Let-7f, 000382). The relative expression of each miRNA was calculated using the comparative Ct (2-ΔΔCt) method.

#### 2.3.4 RNA-sequencing

The NEBNext Ultra II directional RNA library prep kit for Illumina (New England’s Biolabs Inc., Ipswich, MA, USA) was used to prepare mRNA sequencing libraries according to the manufacturer’s instructions. Briefly, 500 ng of total RNA was purified using the NEBNext poly(A) (New Englands Biolabs Inc., Ipswich, MA, USA) and used as a template for cDNA synthesis by reverse transcriptase with random primers. The specificity of the strand was obtained by replacing the dTTP with the dUTP. This cDNA was subsequently converted to double-stranded DNA that was end-repaired. Ligation of adaptors was followed by a purification step with AxyPrep Mag PCR Clean-up kit (Axygen, Big Flats, NY, USA), by an excision of the strands containing the dUTPs and finally, by a PCR enrichment step of 10 cycles to incorporate specific indexed adapters for the multiplexing. The quality of final amplified libraries was examined with a DNA screentape D1000 on a TapeStation 2200, and the quantification was done on the QuBit 3.0 fluorometer (ThermoFisher Scientific, Canada). Subsequently, mRNA-seq libraries with unique index were pooled together in equimolar ratio and sequenced for paired-end 100 pb sequencing on a NovaSeq 6000 flowcell S2 at the Next-Generation Sequencing Platform, Genomics Center, CHU de Québec-Université Laval Research Center, Québec City, Canada. The average insert size for the libraries was 300 bp. The mean coverage/sample was 27M paired-end reads. Post RNA-seq and differential gene expression analysis were performed using Partek Flow software (Partek Incorporated, MO, USA) using a 10-reads cut-off. Pathway analysis was done using Ingenuity Pathway Analysis (IPA) software (Qiagen, CA, USA).

### 2.4 Immunofluorescence

Striatum sections (20 μm) were cut (Leica Microsystems CM1850, Nussloch, Germany). Tissue sections stained with DAPI (nuclei) in cyan, the marker DARPP-32 (dopaminergic neurons) in green and EM48 (targeting aggregated mHTT) in red. Image series of the striatum were taken using a confocal microscope (Axio Observer.Z1; Carl Zeiss, Germany) and acquired using a Quorum WaveFX spinning disk confocal system (Quorum Technologies, Ontario, Canada). Solid state laser lines 491 nm and 561 nm were used for excitation of green and red (Alexa-488 and Alexa-594), combined with appropriate BrightLine single-bandpass emission filters (536/40 nm and 624/40 nm, Semrock, NY, USA). z-series were acquired simultaneously with DAPI fluorescence filter cube (Chroma Technology, VT, USA). The CCD camera used to capture the images was a Hamamatsu Image EM C-9100. Images were acquired and analyzed using Volocity software, version 4.2.1. Iterative restoration (deconvolution) was applied for the DAPI channel, using the same software. The pipeline to quantify HTT inclusions is available upon request.

### 2.5 Open field behavioral test

Behavioral tests were conducted as before (Hernandez-Rapp et al., 2015). In brief, after 30 min of acclimation to the testing room, each mouse was placed in the center of a white box (50 cm^2^) for 10 min, allowing them to explore freely. The box was virtually divided into a center and four corners, and the movements were recorded. Voluntary locomotor activity was measured via general movement in the center compared to the corners. Immobility duration, Freezing time, movement speed, and travel distance in the corners were analyzed using the Any-maze software (Stoelting Co, Wood Dale, IL, United States).

### 2.6 Statistical analysis

All graphics and statistical analyses were performed using GraphPad Prism 9 Software (Graph Pad Software, Inc., La Jolla, CA, USA). Normality tests were performed, and parametric or non-parametric tests were used accordingly (see “3 Results”). When sample distribution passed the normality test, a parametric one-way analysis of variance (ANOVA) test followed by Dunn’s multiple comparisons and parametric unpaired student *t*-test were performed. When sample distribution did not pass the normality test, a non-parametric Kruskal–Wallis test followed by Dunn’s multiple comparisons and a non-parametric Mann-Whitney student *t*-test was performed. The threshold for statistical significance was set to *p* < 0.05.

## 3 Results

### 3.1 miR-132/212 loss correlates with neurodegeneration in the post-mortem human brain

**FIGURE 1:**
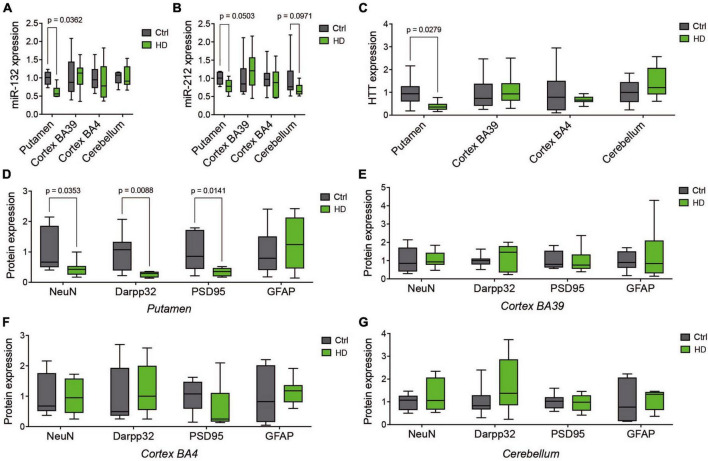
Comparative analysis of human HD brain regions. Relative expression of miR-132 **(A)** and miR-212 **(B)** between human HD putamen, cortex (BA39 & BA4), and cerebellum and Controls as measured by RT-qPCR. **(C)** Western blot quantifications of endogenous full-length HTT **(C)** as well as NeuN, Darpp32, PSD95, and GFAP in the putamen **(D)**, BA39 **(E)**, BA4 **(F)**, and cerebellum **(G)**. Box plots with min-max error bars are shown, where the average of controls was set at 1. *N* = 9–10 per group, mixed sex. Statistics: Ctl vs. HD was calculated using one-way or two-way ANOVA. Significant *P*-values are presented for each group. Ctrl, Controls; HD, Huntington’s disease.

We first sought to investigate in more detail the relationship between miR-132/212 levels and neuronal survival across the human HD brain. Toward this end, we analyzed the putamen, two cortical regions (BA39 and BA4) and the cerebellum from the same individuals (*N* = 10 per group, males and females) in a cohort from the NIH NeuroBioBank (Supplementary Table 1). In these specimens, we observed downregulation of miR-132 (Figure 1A) and miR-212 (Figure 1B) in the putamen, with a trend (*P* = 0.09) for lower miR-212 in the cerebellum. Consistent with earlier findings (St-Amour et al., 2018), we observed lower HTT protein levels in the putamen of affected individuals by Western blot (Figure 1C; Supplementary Figure 1), indicative of HTT neuropathology. Indeed, these changes were associated with a decrease of neuronal (NeuN, Darpp32) and synaptic (PSD95) markers in the putamen (Figure 1D) but not in other brain regions (Figures 1E–G). No changes in GFAP, a marker of inflammation, were observed in all the studied tissues. These results further strengthen the link between miR-132/212 downregulation and neurodegeneration in the post-mortem human HD brain (Petry et al., 2022) but could not disassociate miR-132/212 levels from cell loss *per se*.

### 3.2 Generation of miR-132/212-deficient zQ175 HD mice

We next asked whether miR-132/212 deficiency could influence disease-related phenotypes *in vivo* in HD mice. For this, we crossed miR-132/212 floxed knockout (KO) mice (Magill et al., 2010) with zQ175 knock-in HD mice (Menalled et al., 2012; Southwell et al., 2016). These latter mice have the murine Htt exon 1 replaced with the human HTT exon 1 sequence with ∼180–220 CAG repeats. Heterozygote zQ175 mice develop classical HD-related features, including motor deficits, HTT inclusions, and neurodegeneration (Menalled et al., 2012; Carty et al., 2015; Southwell et al., 2016; Jansen et al., 2017; Smith et al., 2023). To avoid potential compensation mechanisms during development, we used an inducible KO approach to specifically target miR-132/212 in adult mice using a Cre-ERT2 strain (See Methods). Permanent miR-132/212 gene deletion was induced with tamoxifen at 2 months of age. We generated four experimental groups of mice for this study, namely, wildtype (WT), miR-132/212 KO (KO), zQ175 (HD), and miR-132/212 KO; zQ175 (HD/KO) on the same genetic background.

**FIGURE 2:**
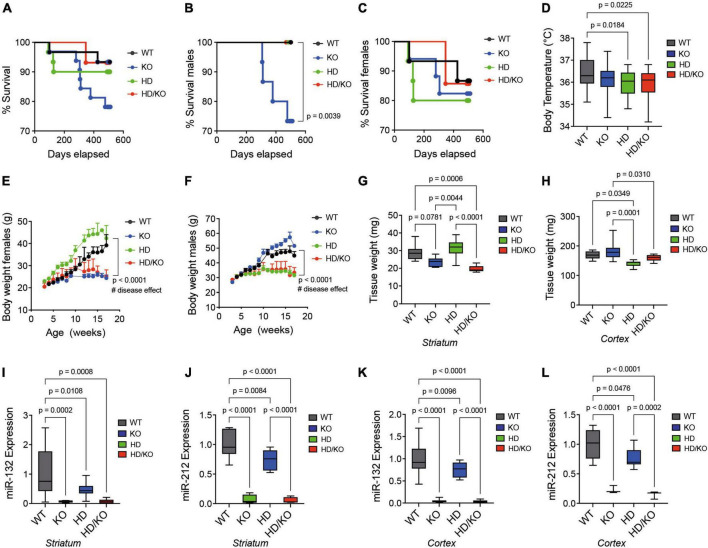
Characterization of a novel miR-132/212-deficient HD mouse model. Kaplan-Meier survival plot of all groups of mice **(A)** combined or separated by **(B)** males or **(C)** females (*N* = 14–15 per sex). **(D)** The rectal temperature of mice was taken at sacrifice at 18 months of age (*N* = 23–25 per group, mixed sex). Body weight differences in **(E)** females and **(F)** males between the groups of mice during aging (*N* = 10–17 per sex). The post-mortem quantifications of wet brain tissue weight in the mouse **(G)** striatum and **(H)** cortex immediately after sacrifice. Brain regions were snap-frozen on dry ice before measurements (*N* = 6–9 per group, mixed sex). Relative expression of miR-132 and miR-212 mouse striatum **(I,J)** and cortex **(K,L)** as measured by RT-qPCR (*N* = 10 per group, mixed sex). Box plots with min-max error bars are shown, where the average of controls was set at 1. Statistics: Differences in survival were calculated using a simple survival analysis (Kaplan-Meier). For body weights, one-way ANOVA with Brown-Forsythe and Welch test was performed. Others were calculated using a one-way ANOVA. Significant *P*-values are presented for each group. WT, wildtype; KO, knockout; HD, Huntington’s disease, HD/KO, Huntington’s disease/knockout.

A Kaplan-Meier plot showed no differences in survival among all the groups of mice tested up until sacrifice at 18 months of age (Figure 2A). Interestingly, however, the survival curves of single KO males (Figure 2B), when separated from single KO females (Figure 2C), were significantly lower. This effect was not seen in double HD/KO mice. Consistent with previous findings (Weydt et al., 2006), body temperature was lower in HD mice at sacrifice (Figure 2D). In addition, progressive weight loss was observed in HD males (Figure 2E) and HD females (Figure 2F), as seen before (Menalled et al., 2012; Southwell et al., 2016). However, there was no additive effect of miR-132/212 deletion on body temperature or body weight. No other sex-related differences were observed throughout this study. The postmortem analysis of brain weight showed significant differences among regions and genotypes (Figures 2G, H). These experiments focused on the striatum and the cortex, two regions decorated with HTT inclusions in the zQ175 model (Carty et al., 2015; Jansen et al., 2017; Smith et al., 2023). By RT-qPCR, we could confirm that both miR-132 and miR-212 were absent in the KO and HD/KO mice in the striatum (Figures 2I, J), cortex (Figures 2K, L), and other brain regions tested, such as the cerebellum, brain stem, and olfactory bulb (not shown). These results demonstrate that the zQ175 HD phenotypes recapitulate the literature well in our hands, while global deletion of miR-132/212 in adult HD mice is viable and tolerated.

### 3.3 Partial improvement of HD-related motor function following miR-132/212 deletion

**FIGURE 3:**
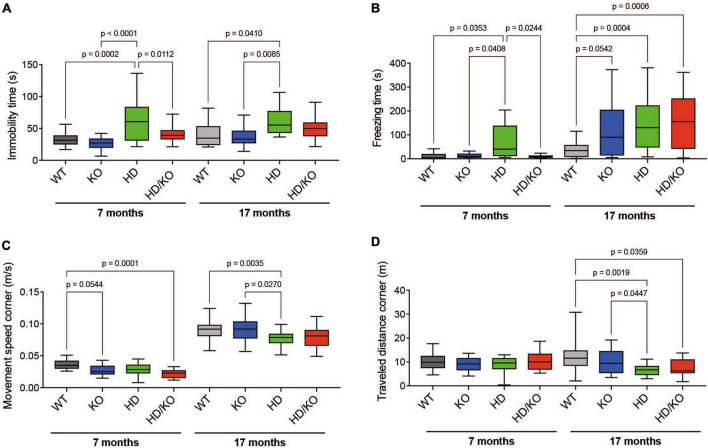
Assessment of motor function in HD zQ175 mice lacking miR-132/212. An open-field motor test was performed on all groups of mice at 7 and 17 months. Automated open-field activity was measured in all groups of mice for **(A)** immobility time, **(B)** freezing time, **(C)** movement speed in corners, and **(D)** travel distance in corners. Box plots with min-max error bars are shown, where the average of controls was set at 1. *N* = 13–28 per group, mixed sex. Statistics were calculated using a one-way Brown-Forsythe and Welch ANOVA test. Significant *P*-values are presented for each group. WT, wildtype; KO, knockout; HD, Huntington’s disease, HD/KO, Huntington’s disease/knockout.

All mice were also evaluated for behavioral changes during aging. For this, we used an open-field motor test in which heterozygote zQ175 mice become deficient at 2–6 months of age (Menalled et al., 2012; Southwell et al., 2016). Here, the same mice were tested at 7 and 17 months. As previously reported (Hernandez-Rapp et al., 2015), no major changes in open-field activities were observed in single KO mice. On the other hand, as expected, HD mice performed poorly in behavioral tests, especially in aged mice. Interestingly, at 7 months of age, miR-132/212 deletion restored immobility time (Figure 3A) and freezing time (Figure 3B) in HD mice but not movement speed (Figure 3C) or total distance traveled (Figure 3D). No significant effects of miR-132/212 abrogation were observed in other events (e.g., total movement speed) (not shown). These results suggest that miR-132/212 gene deletion provides a partial and temporary benefit in HD-related behavior in mice.

### 3.4 MiR-132/212 deficiency partially rescues HD-related pathologies in aged mice

**FIGURE 4:**
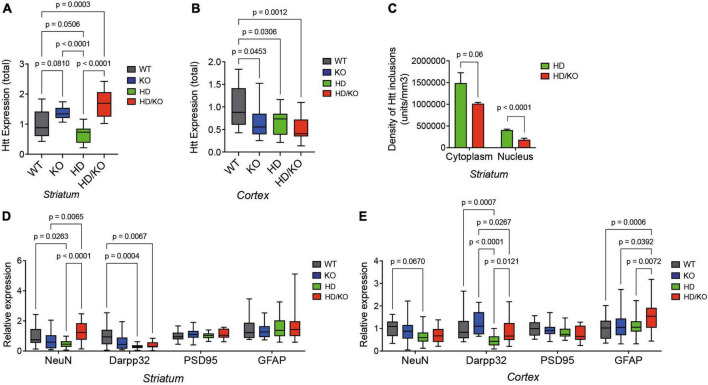
Characterization of HD pathology in the absence of miR-132/212. **(A)** Western blot quantifications of full-length total Htt expression in the **(A)** striatum and **(B)** cortex of experimental mouse groups (*N* = 14–16 per group, mixed sex). **(C)** Digital quantifications of mHTT inclusions separated by subcellular localization (five images per section using *N* = 3–4 mice per group, mixed sex). Western blot quantifications of endogenous NeuN, Darpp32, PSD95, and GFAP in the putamen **(D)** and cortex **(E)** of different mouse groups (*N* = 17–23 per group, mixed sex). Box plots with min-max error bars are shown, where the average of controls was set at 1. Statistics were calculated using two-way ANOVA. Significant *P*-values are presented for each group. WT, wildtype; KO, knockout; HD, Huntington’s disease, HD/KO, Huntington’s disease/knockout.

A postmortem analysis of brain tissues revealed a lower expression of total Htt protein levels in the striatum (Figure 4A) and cortex (Figure 4B) of HD mice at sacrifice, as seen in humans. Interestingly, this effect was abolished in the striatum of HD/KO mice. Note that, however, human mutant HTT (mHTT) protein levels were not altered between HD and HD/KO mice (Supplementary Figure 2). Furthermore, a dot blot analysis of Htt/mHTT protein aggregation following formic acid treatment showed no significant differences between HD and HD/KO mice (not shown). However, the detailed quantification of mHTT protein inclusions by immunofluorescence (Supplementary Figure 3) showed fewer aggregates in the cytoplasm and nucleus of striatal neurons in HD/KO compared to HD mice (Figure 4C). Additional experiments using NeuN and Darpp32 showed a partial rescue of neuronal and synaptic integrity in the striatum and cortex upon miR-132/212 depletion in HD/KO mice (Figures 4D, E). Taken together, these results suggest that miR-132/212 deficiency restores, at least in part, HD-related phenotypes at the biochemical level in aged mice.

### 3.5 Enrichment of HD-related pathways and gene targets upon miR-132/212 deletion in the brain

**FIGURE 5:**
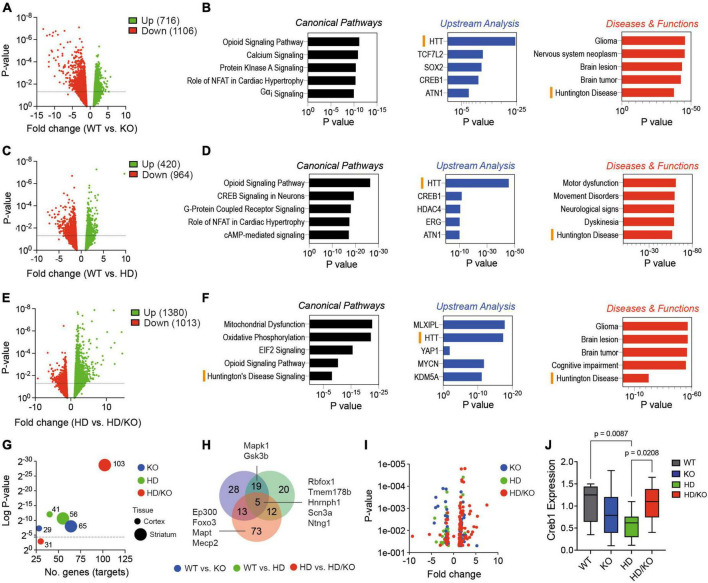
Transcriptomics analysis of miR-132/212-deficient HD mice. **(A,C,E)** Volcano plots of differently expressed genes (DEG) in the striatum of selected mouse genotypes (*N* = 5 brain samples per group). The numbers of significant (*P* < 0.05, 10 reads cut-off, all fold changes) respective upregulated and downregulated genes are indicated. **(B,D,F)** IPA-generated pathways are shown. Both upregulated and downregulated genes were included in the analyses. Significant biological functions include HTT, Huntington’s disease and Creb signaling in neurons. **(G)** The MEINETURNET tool was used to identify miR-132/212 gene targets in the DEG datasets. For comparative reasons, we included significantly (*P* < 0.05) misregulated transcripts from the mouse striatum and cortex. **(H)** Venn diagram and Volcano plot **(I)** of the identified miR-132/212 targets in the striatum. **(J)** Western blot quantifications of endogenous Creb1 in the striatum of experimental mice. Box plots with min-max error bars are shown, where the average of controls was set at 1. Statistics were calculated using two-way ANOVA. Significant *P*-values are presented for each group. WT, wildtype; KO, knockout; HD, Huntington’s disease, HD/KO, Huntington’s disease/knockout. Orange bars highlight common HD-related pathways.

We finally performed a transcriptomics analysis to understand better the genes and pathways modulated by miR-132/212 deficiency. We isolated total RNA from postmortem brain tissues and performed mRNA-seq. Volcano plot analysis showed differential gene (transcript) expression in striatal tissues according to mouse genotypes (WT vs. KO; WT vs. HD; HD vs. HD/KO) (Figures 5A, C, E). We observed little overlap in differently expressed genes (DEG) between miR-132/212 KO backgrounds (Supplementary Figure 4). Pathway analysis using the Ingenuity Pathway Analysis (IPA) software was next conducted using the genes that were significantly (*P* > 0.05) modulated among the groups of mice (Figures 5B, D, F; Supplementary Table 3). Interestingly, substantial overlap was observed at the network level, including terms such as *Huntington’s disease* and *HTT* in KO, HD, and HD/KO mice. Other shared pathways include the *Opioid Signaling Pathway* and *Creb1*. As expected, HD mice were strongly associated with *Motor dysfunction* and *Movement disorders*. Interestingly, miR-132/212 deletion in HD mice was associated with unique pathways such as *Mitochondrial dysfunction* and *Oxidative Phosphorylation*.

Next, we used the MIENTURNET online tool (Licursi et al., 2019) to study miRNA target enrichment among the significant (*P* > 0.05) differently expressed genes. Interestingly, the highest number of miR-132/212 targets (103 in total) were found in HD/KO compared to HD mice (Figure 5G). This effect was most prominent in the striatum when compared to the cortex. A Venn diagram shows the overlap of miR-132/212 targets between groups (Figure 5H). Of interest, known miR-132/212 targets, such as Mapt, Creb1, Mapk1/Erk2, Mecp2, Ep300 and Foxo3, were found exclusively in HD/KO mice. As expected, most targets were upregulated following miR-132/212 deletion in the latter group (Figure 5I). Finally, we observed a significant downregulation of Creb1 protein levels, a key component of the BDNF-miR-132/212 axis in HD but not HD/KO mice (Figure 5J). These effects correlated with changes in mature BDNF protein levels (Supplementary Figure 5). Taken together, these results suggest that miR-132/212 deletion in adults promotes distinct changes in gene expression patterns in either normal or pathological conditions.

## 4 Discussion

In this study, we investigated for the first time the effects of miR-132/212 deficiency in a mouse model of HD. This approach was prompted by the observation that miR-132 and miR-212 are consistently downregulated in human HD brain. Consistent with previous observations (Magill et al., 2010; Remenyi et al., 2013; Hernandez-Rapp et al., 2015, 2016; Smith et al., 2015; Hansen et al., 2016), gene depletion of the miR-132/212 cluster was viable and well tolerated in mice. However, significant changes in mouse behavior, pathological markers, and gene expression profiles suggest a contributing or compensatory role for miR-132/212 in HD onset or progression.

As confirmed herein, miR-132/212 expression is lower in HD subjects compared to non-affected controls. We also provide the first comparative study of four brain regions (putamen, BA39, BA4, cerebellum) from the same individuals in humans. In contrast to our previous studies (St-Amour et al., 2018; Petry et al., 2022), the brain samples herein were not classified by Vonsattel disease stage. However, we suppose that most, if not all, tissues were isolated from early and mid-stage disease (i.e., HD2 and HD3) as no overt signs of neurodegeneration were seen in the cortical and cerebellar regions. This could explain the relatively small changes in miR-132 and miR-212 expression in the putamen compared to previous observations. Nevertheless, these specimens provide a unique model to study the cause-or-effect relationship between miR-132/212 expression and disease within the same individual. Indeed, the selected brain regions are differently affected during the disease course (putamen > BA4 > BA39 > cerebellum). While interesting, these observations could not help us determine if the loss of miR-132/212 *per se* could promote neurodegeneration *in vivo*.

For this reason, we generated zQ175 heterozygote mice lacking the miR-132/212 cluster. Since miR-132/212 is expressed at early stages of brain development (Smith et al., 2011), we used an inducible knockout approach to remove miR-132/212 in young adults. While grossly normal, one exception was noted where single KO male mice displayed lower survival rates than females. This phenomenon is novel and seems specific to breeding with Cre-ERT2 mice. While further investigation is required, this observation could implicate sex- or hormone-specific miR-132/212 targets such as Nurr1 (Yang et al., 2012). Another interesting observation was the effects on wet brain weight vs. neuronal markers like NeuN. It could be that miRNA loss promotes, in addition to neurodegeneration, cellular shrinkage (Hebert et al., 2010) or implicates other cell types. A detailed stereological study would be required to address this issue.

In this study, we focused mainly on the mouse striatum and, to a lesser extent, the cortex for detailed biochemical and transcriptional analyses. This was motivated by the fact that HD pathology in humans is predominantly located in the striatum and, to a lesser degree, the cortex in late-stage disease. Of course, zQ175 mice display high levels of HTT inclusions and degeneration in both brain regions [(Carty et al., 2015; Jansen et al., 2017; Smith et al., 2023) and results herein]. This said, the overall effects of miR-132/212 deficiency in the striatum and cortex of zQ175 mice if directly compared to humans, need to be interpreted with some caution. In addition, miR-132/212 gene depletion occurs early in adulthood and in all other brain regions and peripheral tissues, some of which could theoretically contribute to HD-related pathologies. One example includes the brain-heart axis that goes awry in HD (Mielcarek et al., 2014; Critchley et al., 2018). Interestingly, miR-132/212 deletion also impacts heart size and cardiac function in mice (Ucar et al., 2012; Lei et al., 2020). Future studies will require striatal-specific deletion of miR-132/212 in adult or aged mice using, for instance, an inducible Cre recombinase under the control of the Gpr88 promoter.

The most surprising and unexpected aspect of this study involves the seemingly partial benefits of miR-132/212 loss on HD-related pathologies and behavior in mice. Indeed, prior studies showed that removing the miR-132/212 cluster from transgenic mouse models of neurodegenerative disease (i.e., Alzheimer’s disease and Tauopathies) (Hernandez-Rapp et al., 2016; Boscher et al., 2020) exacerbated disease phenotypes. Artificially modifying miR-132 levels using oligonucleotides resulted in similar outcomes: worsening or improving disease-related phenotypes after miR-132 inhibition or overexpression, respectively (Salta et al., 2014, 2016; El Fatimy et al., 2018; Xu et al., 2019; Su et al., 2020). Notably, miR-132 supplementation rescued motor deficits (but not HTT pathologies) in HD mice (Fukuoka et al., 2018). Our current observations are puzzling and suggest a robust compensation mechanism between miR-132/212 (downregulation) and its targets in the context of neurodegeneration. Dose-response and feedback relationships do exist between miR-132/212, BDNF (Keifer et al., 2015), and Creb1 (Hollander et al., 2010; Hansen et al., 2012), both of which are expressed normally in HD/KO compared to HD mice. Interestingly, the Creb1 signaling pathway is a potent modulator of neurodegeneration and is linked to HD (Mantamadiotis et al., 2002; Jeong et al., 2011; Aravindan et al., 2020; Seefelder and Kochanek, 2021). The miR-132/212-Creb1 axis also controls hormone-related functions, including corticosterone modulation of synaptic plasticity (Kouhnavardi et al., 2023). Correlation studies between neurotrophins and other factors with miR-132/212 expression at different ages are, therefore, warranted, including in the human HD brain.

Of course, the fact that miR-132/212 deletion did not fully rescue HD-related phenotypes over time, even with seemingly lower HTT inclusions at sacrifice, implies additional factors and age-related mechanisms are at play. These observations also support previous observations dissociating HTT inclusions with neurodegeneration (Gutekunst et al., 1999; Gan et al., 2018; Hickman et al., 2022). In the future, it would be interesting to map HTT pathologies throughout the brain and perhaps other organs in these mice. Also, performing additional behavioral tasks related to motor function (e.g., grip strength) and cognition (e.g., novel object recognition, Barnes maze) at all stages of the disease would inform us on the interplay between miR-132/212 and Htt on the functional phenotype.

The identification of miR-132/212 physiological targets remains an ongoing challenge. Previous studies using genetic, pharmacological, or biochemical methods have yet to reach a consensus, and multiple technical and biological variables must be considered (Boscher et al., 2020; Krichevsky et al., 2023; Walgrave et al., 2023). Despite this, a handful of putative miR-132/212 targets have emerged from gain and loss-of-function studies *in vivo*. These include, in addition to Creb1, Mapt, Mapk1/Erk2, and Mecp2 (Boscher et al., 2020; Zhang and Bian, 2021) which were also identified in this study, at least at the mRNA levels. These targets are likely stimulated by context-dependent factors such as neurotrophins or hormones. The fact that most miR-132/212 targets become exclusively upregulated in HD/KO mice supports this hypothesis and is somewhat consistent with the neuroprotective role of miR-132 (Weinberg et al., 2015; El Fatimy et al., 2018; Krichevsky et al., 2023). An often overlooked issue is the potential unique target(s) and role(s) of miR-132 and miR-212 *in vivo* (Hansen et al., 2016). The primary algorithm used in this study, TargetScan, does not distinguish miR-132/212 targets since the predictions are based on the conserved (shared) seed sequences. It will be interesting to study miRNA-specific KO mouse models in the future to address this important issue. Finally, mounting studies support the view of cell-specific regulation of miR-132/212 targets *in vivo* (Walgrave et al., 2023).

Interestingly, our network analyses provided insight into the molecular mechanisms behind miR-132/212-induced benefits. In this regard, two biological pathways related to mitochondria and oxidative stress stood out specifically in HD mice, both of which are interconnected and have known implications in HD pathology (Zheng et al., 2018; Dai et al., 2023). Interestingly, miR-132 was linked to mitochondria or oxidative stress pathways in other contexts (Weinberg et al., 2015; Liu et al., 2022). A functional link also exists between mitochondria, oxidation, and neurotrophic effectors like Creb1 (Pregi et al., 2017). Therefore, whether BDNF signaling is the sole mediator of miR-132/212-dependent “rescue” is unclear. Interestingly, miR-132/212 deletion is sufficient to promote HTT and HD-related pathways in single KO mice, further suggesting a strong functional link, direct or indirect, between these two factors. On this line of thought, future studies are required to address the apparent divergent effects of miR-132/212 loss on mouse Htt vs. human mHTT (e.g., expression, inclusions).

In conclusion, this study provides important additional clues into the *in vivo* function of miR-132/212 in the brain. The precise molecular mechanism leading to miR-132/212 downregulation in the HD brain remains unsettled and likely involves multiple cues or signaling pathways. Prominent candidates include BDNF effectors such as Creb1, but also Mapk/ERK, Mecp2 and others identified herein. In a previous report, we observed important maturation impairments of the miR-132/212 transcript in human HD (Petry et al., 2022). This study was not designed to address the role of miRNA maturation impairments in HD mice. In the future, it would be relevant to measure miR-132/212 expression and maturation levels in the prodromal and very early stages of HD. In mice, the specific deletion of miR-132/212 in adult striatal cells warrants further investigation. In a more general sense, we provide further evidence that small regulatory non-coding RNAs such as miR-132/212 (representing about 0.25–1% of total miRNAs in the adult brain) could greatly impact brain health and disease.

## Data availability statement

The data presented in the study are deposited in the GEO repository, accession number GSE84481.

## Ethics statement

The studies involving humans were approved by CHU de Québec Human Research Ethics Committee. The studies were conducted in accordance with the local legislation and institutional requirements. The ethics committee/institutional review board waived the requirement of written informed consent for participation from the participants or the participants’ legal guardians/next of kin because consents were initially obtained by the NIH NeuroBioBank. The animal study was approved by Comité de Protection des Animaux de l’Université Laval (CPAUL). The study was conducted in accordance with the local legislation and institutional requirements.

## Author contributions

BN: Conceptualization, Data curation, Formal analysis, Investigation, Methodology, Validation, Visualization, Writing – original draft, Writing – review & editing. RK: Data curation, Formal analysis, Investigation, Methodology, Software, Writing – review & editing. GB: Writing – review & editing. MaB: Data curation, Formal analysis, Methodology, Software, Visualization, Writing – review & editing. CG: Investigation, Methodology, Writing – review & editing. GC: Investigation, Methodology, Writing – review & editing. AL: Writing – review & editing. IS-A: Conceptualization, Data curation, Formal analysis, Investigation, Methodology, Supervision, Validation, Writing – review & editing. EP: Resources, Writing – review & editing. DS: Conceptualization, Data curation, Formal analysis, Resources, Software, Writing – review & editing. SH: Conceptualization, Formal analysis, Funding acquisition, Methodology, Project administration, Resources, Supervision, Visualization, Writing – original draft.
