# Supplementary material for: Beneficial effects of miR-132/212 deficiency in the zQ175 mouse model of Huntington’s disease
Source: Front Neurosci. 2024 Aug 7;18:1421680. doi: 10.3389/fnins.2024.1421680 (PMC11337869; doi:10.3389/fnins.2024.1421680)
Supplement: Supplementary file 1 [file Data_Sheet_1.PDF]

Supplementary Material

Beneficial effects of miR-132/212 deficiency in the zQ175 mouse model of Huntington's disease

Behnaz Nateghi[1, 2, #], Remi Keraudren[1, 2, #], Gabriel Boulay[1, 2], Mohamed Lala Bouali[1, 2], Marc Bezin[1], Claudia Goupil[1], Geoffrey Canet[1], Andréanne Loiselle[1], Isabelle St-Amour[3], Emmanuel Planel[1, 2], Denis Soulet[1, 4], Sébastien S. Hébert[1, 2]

*** Correspondence:** Dr. Sébastien S. Hébert: sebastien.hebert@crchudequebec.ulaval.ca

1 Supplementary Figures and Tables

1.1 Supplementary Figures 1-5
1.2 Supplementary Tables 1 and 2
1.3 Supplementary Table 3 (see additional Excel file)

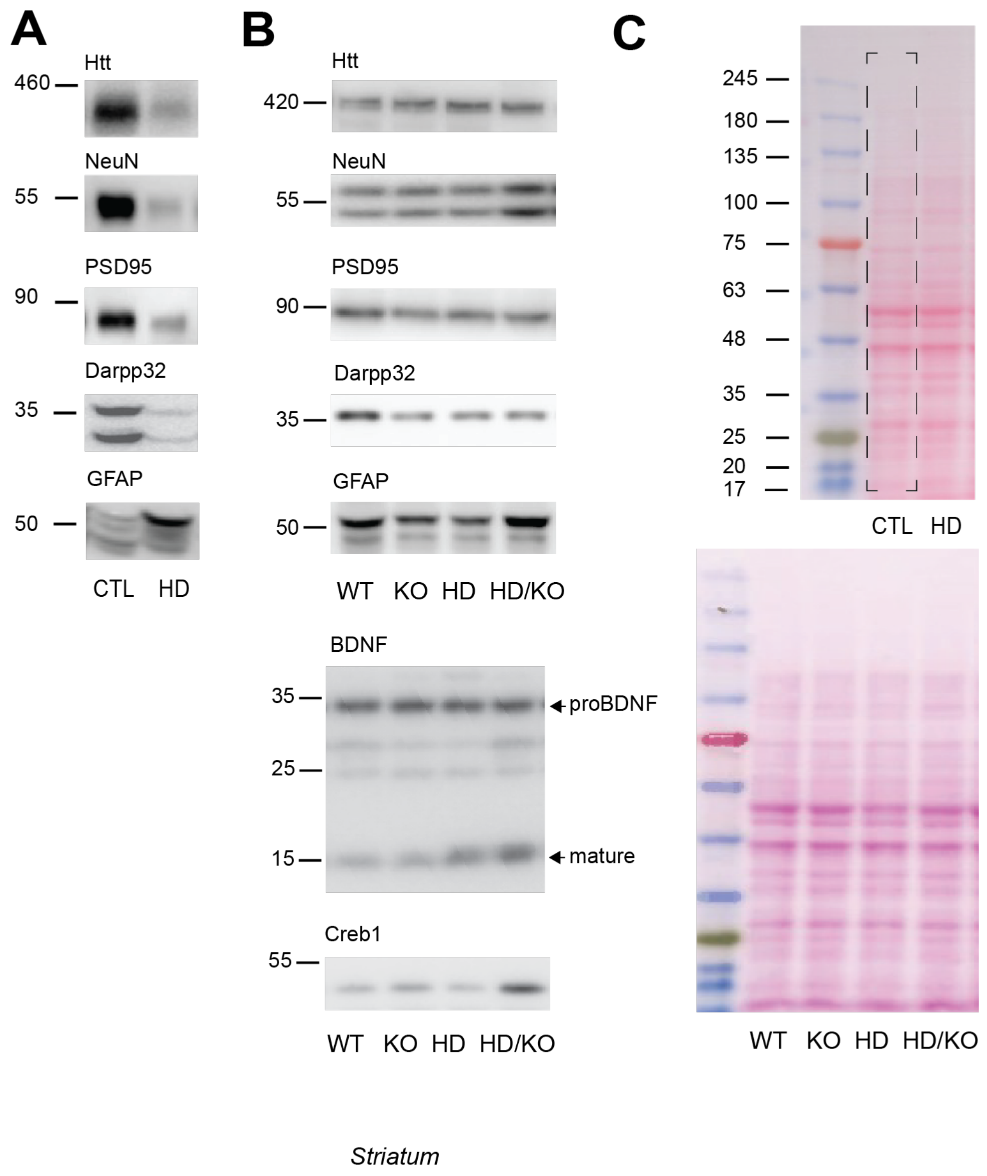

Supplementary Figure 1. (A) Representative Western blot images of endogenous Htt, PSD95, NeuN, Darpp32, and GFAP in the human putamen. **(B)** Representative Western blot images of endogenous Htt, PSD95, NeuN, Darpp32, GFAP, BDNF and Creb1 in the mouse striatum. In each experiment, quantifications were normalized to total proteins (e.g., Ponceau as in **(C)** and/or Stain-Free). Please note that not all blots were run at the same time or gel systems. Similar band sizes were observed in the other brain regions. A representative square is shown in C, which denotes the region used for normalizations. Band size(s) in kDa are shown. Abbreviations: WT, wildtype; KO, knockout; HD, Huntington's disease, HD/KO, Huntington's disease/knockout.

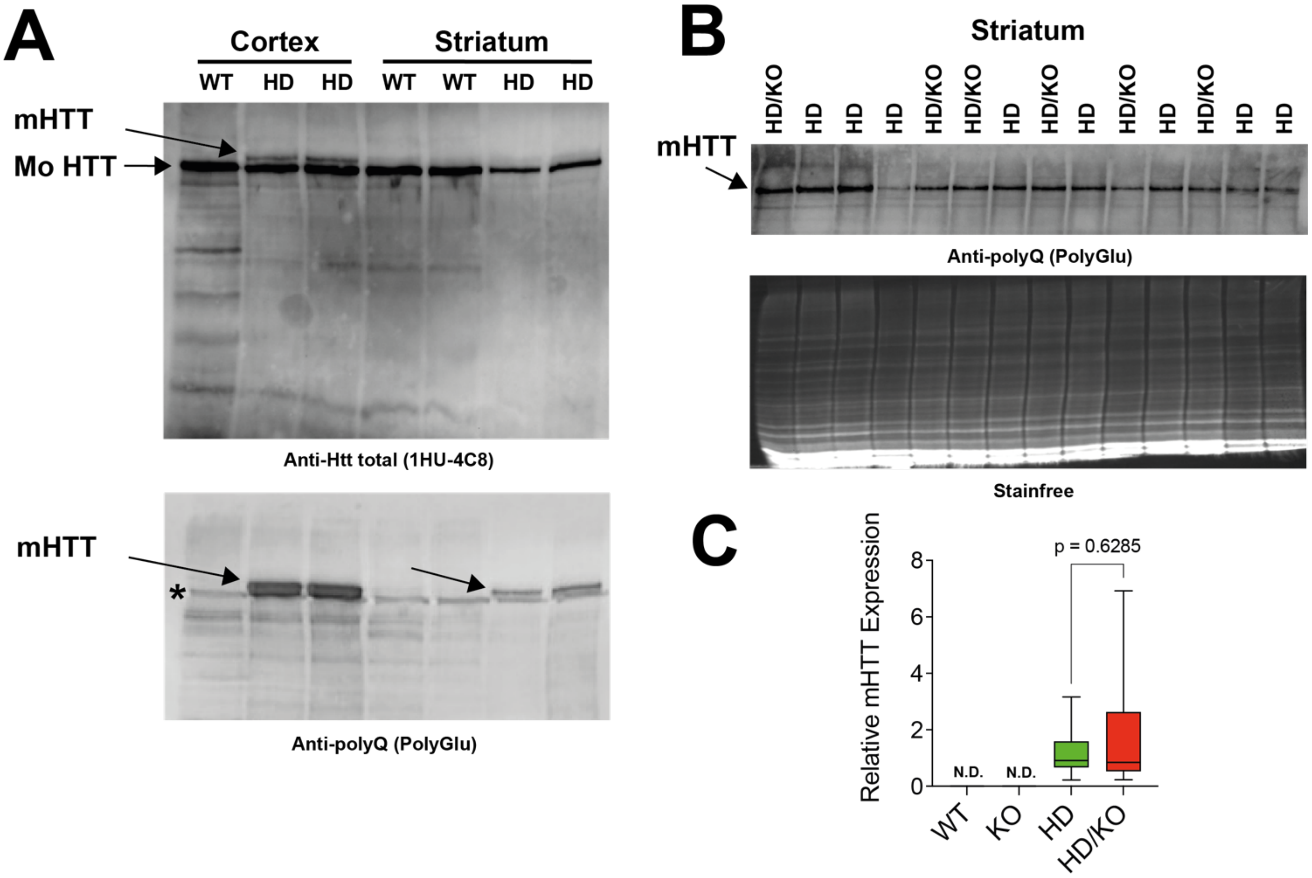

Supplementary Figure 2. (A) Representative Western blot images of Htt using two different antibodies directed against total Htt/mHTT (1HU-4C) and human mHTT (Poly-Glu). Note that the "*" sign represents the remaining mouse Htt signal after membrane stripping. Note that human mHTT is expressed higher in the cortex when compared to the striatum. These examples were taken from adult WT mice (N=1-2 per group) **(B)** The PolyQ antibody is specific to human mHTT. A representative example is shown, where HTT levels, a single band, were normalized to total proteins (Stainfree). **(C)** Quantifications of mHTT in HD and HD/KO mice (N=20-21 samples per group, mixed sex). No significant differences were noted between groups. Box plots with min-max error bars are shown, where the average of controls (HD) was set at 1. Statistics were calculated using a t-test. Abbreviations: WT, wildtype; KO, knockout; HD, Huntington's disease, HD/KO, Huntington's disease/knockout.

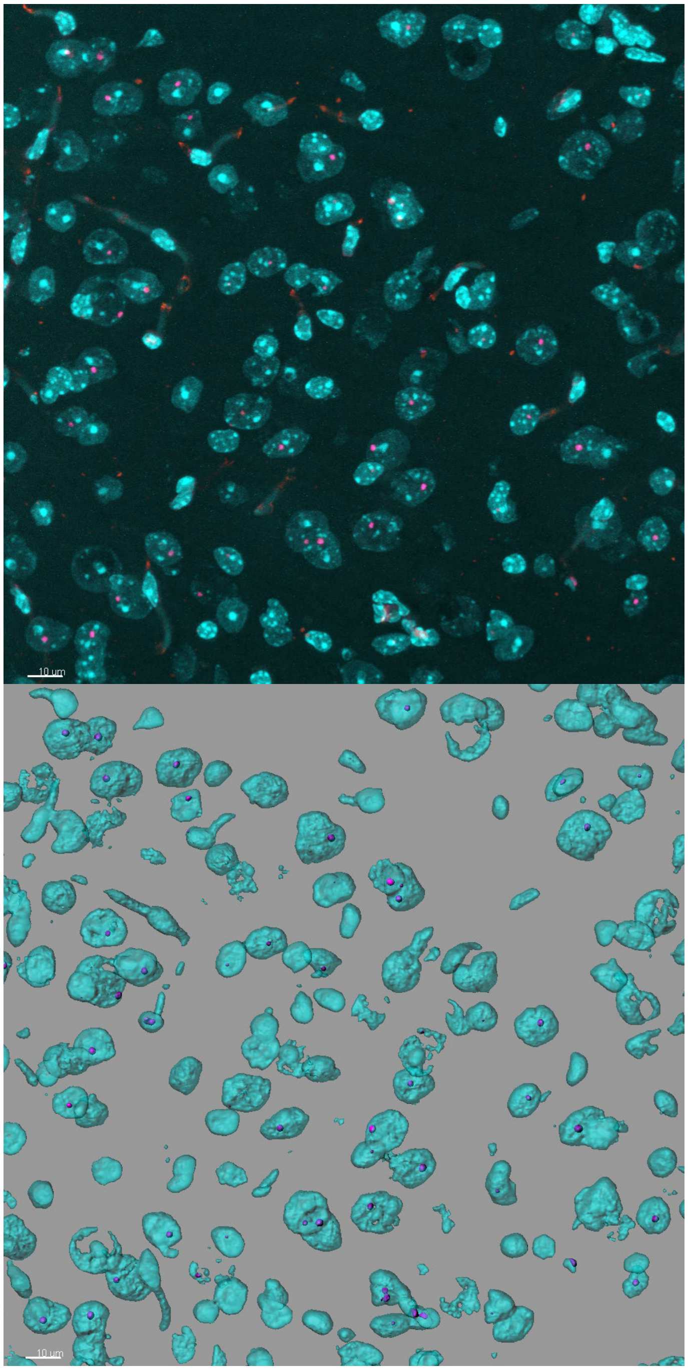

Supplementary Figure 3. HTT inclusion quantifications. (Upper panel) Representative images of cytoplasmic mHTT inclusions (in red) and nuclear mHTT inclusions (in magenta) used for non-biased quantifications in the mouse striatum using the mEM48 antibody. Nuclei are stained with DAPI (in turquoise). (Lower panel) Computerized image showing isolated HTT inclusions in the nuclei. In this example, the area represents 0.6 mm^3 with 57 nuclear inclusions calculated with our algorithm. Non-transgenic WT or KO mice showed no HTT inclusions with the same antibody (not shown). White bar graphs indicate 10 μm.

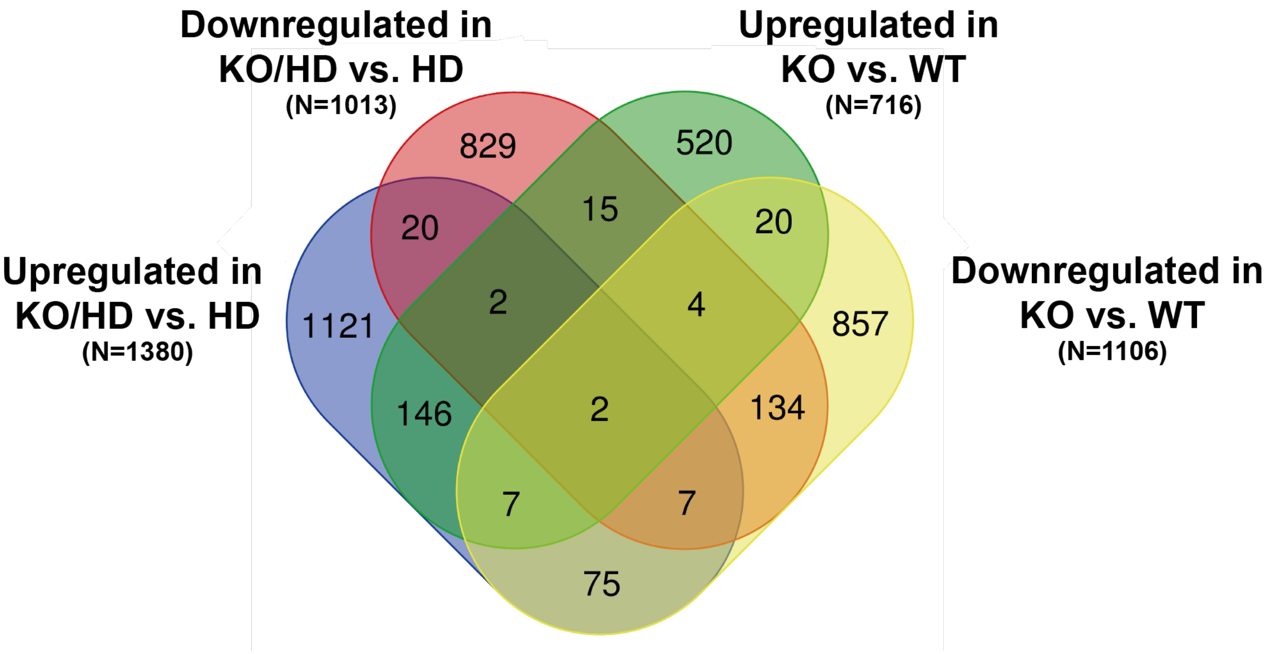

Supplementary Figure 4. Gene alterations according to miR-132/212 knockout background. Venn diagram using significantly (P<0.05, 10 reads and above) altered genes in KO vs. HD/KO mouse DEG datasets used in Figure 5A and E. Up- and down-regulated genes (transcripts) are shown separately. Overall, only 146 upregulated and 134 downregulated genes overlap between knockout models. This observation is consistent with variable putative miR-132/212 targets between backgrounds. N: Number of unique genes (transcripts) in each group.

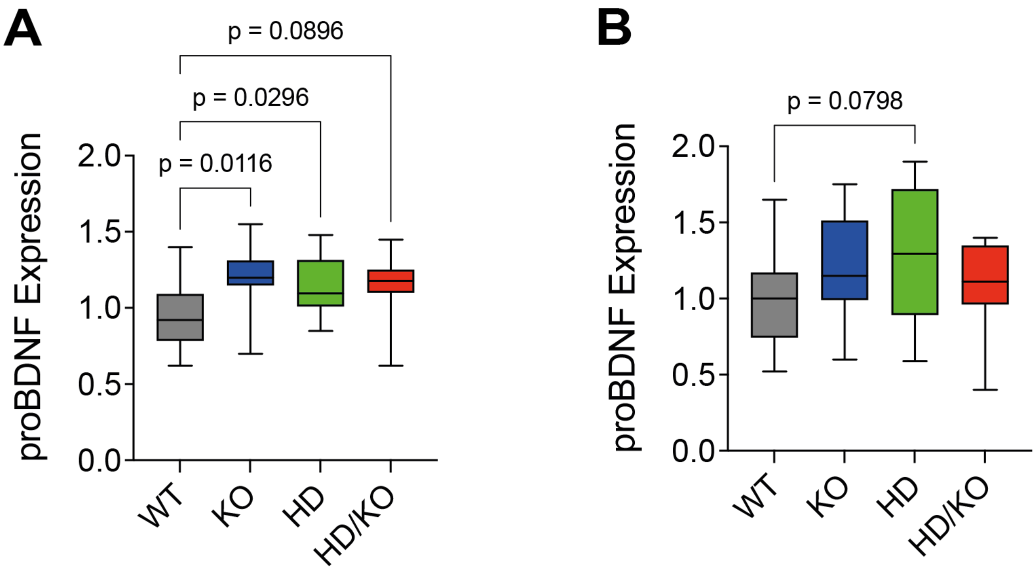

Supplementary Figure 5. BDNF expression analysis. Western blot quantifications of endogenous **(A)** proBDNF (32 kDa) and **(B)** mature BDNF (15 kDa) in the striatum of the different mouse groups (N=11-20 per group, mixed sex). Box plots with min-max error bars are shown, where the average of controls was set at 1. Statistics were calculated using two-way ANOVA. Significant P-values are presented for each group. Abbreviations: WT, wildtype; KO, knockout; HD, Huntington's disease, HD/KO, Huntington's disease/knockout.

	N	Age	PMI	Women (%)	Men (%)
**Control**	10	81 (54-97)	15 (5-48)	70	30
**HD**	10	58 (32-76)	17 (8-22)	60	40

Supplemental Table 1. Characteristics of Huntington's patients and healthy individuals. N, Number; PMI, Post-mortem interval. Age and PMI ranges are shown in brackets.

Name	Protein	Epitope/ immunogen	Clone	Species	Provider & Cat. Number	Dilution
DARPP-32	Dopamine and cyclic AMP-regulated phosphoprotein, relative molecular mass 32000	Residues surrounding Glu160 of human DARPP-32	19A3	Rabbit	Cell Signaling (2306)	1000
GFAP	Glial fibrillary acidic protein		Polyclonal	Rabbit	Thermo Fisher Scientific (PA1-9565)	5000
mEM48	Huntingtin	First 256 a.a. with deletion of polyQ tract	mEM48	Mouse	EMD Millipore (Mab5374)	500
1HU-4C8	Huntingtin	a.a 181-810	1HU-4C8	Mouse	EMD Millipore (Mab2166)	1000
PolyQ	Polyglutamine-Expansion	Poly-Glu	5TF1-1C2	Mouse	EMD Millipore (MAB1574)	1000
NeuN	Neuronal nuclear protein		E4M5P	Mouse	Cell Signaling (4403)	1000
PSD-95	Post-synaptic density protein 95		Polyclonal	Rabbit	Cell Signaling (2507)	2000
BDNF	Brain-derived neurotrophic factor	A peptide mapping within an internal region of BDNF of human origin	Polyclonal	Rabbit	Santa Cruz (N-20)	1000
CREB	CAMP Responsive Element Binding Protein		Polyclonal	Rabbit	Cell Signaling (D76D11)	500

Supplemental Table 2. Antibodies used in this study.
